# Association between the triglyceride glucose-body mass index and future cardiovascular disease risk in a population with Cardiovascular-Kidney-Metabolic syndrome stage 0–3: a nationwide prospective cohort study

**DOI:** 10.1186/s12933-024-02352-6

**Published:** 2024-08-07

**Authors:** Weipeng Li, Chaonan Shen, Weiya Kong, Xiaohui Zhou, Huimin Fan, Yuzhen Zhang, Zhongmin Liu, Liang Zheng

**Affiliations:** 1 Shanghai Heart Failure Research Center, Shanghai East Hospital, Tongji University School of Medicine, Shanghai, China; 2 Research Center for Translational Medicine, Shanghai East Hospital, Tongji University School of Medicine, Shanghai, China; 3 Department of Epidemiology and Public Health, Tongji University School of Medicine, Shanghai, China; 4 School of Public Health, Medical College of Soochow University, Suzhou, China; 5 Institute of Integrated Traditional Chinese and Western Medicine for Cardiovascular Chronic Diseases, Tongji University School of Medicine, Shanghai, 200120 China

**Keywords:** Cardiovascular kidney metabolic syndrome, CVD, IR, TyG-BMI

## Abstract

**Background:**

The American Heart Association (AHA) has recently introduced the concept of Cardiovascular-Kidney-Metabolic (CKM) syndrome, which is the result of an increasing emphasis on the interplay of metabolic, renal and cardiovascular diseases (CVD). Furthermore, there is substantial evidence of a correlation between the triglyceride glucose-body mass index (TyG-BMI ) and CVD as an assessment of insulin resistance (IR). However, it remains unknown whether this correlation exists in population with CKM syndrome.

**Methods:**

All data for this study were obtained from the China Health and Retirement Longitudinal Study (CHARLS). The exposure was the participants’ TyG-BMI at baseline, which was calculated using a combination of triglycerides (TG), fasting blood glucose (FBG) and body mass index (BMI). The primary outcome was CVD, which were determined by the use of a standardised questionnaire during follow-up. To examine the relationship between TyG-BMI and CVD incidence in population with CKM syndrome, both Cox regression analyses and restricted cubic spline (RCS) regression analyses were performed.

**Results:**

A total of 7376 participants were included in the final analysis. Of these, 1139, 1515, 1839, and 2883 were in CKM syndrome stages 0, 1, 2, and 3, respectively, at baseline. The gender distribution was 52.62% female, and the mean age was 59.17 ± 9.28 (years). The results of the fully adjusted COX regression analyses indicated that there was a 6.5% increase in the risk of developing CVD for each 10-unit increase in TyG-BMI,95% confidence interval (CI):1.041–1.090. The RCS regression analyses demonstrated a positive linear association between TyG-BMI and the incidence of CVD in the CKM syndrome population (P for overall < 0.001, P for nonlinear = 0.355).

**Conclusions:**

This cohort study demonstrated a positive linear association between TyG-BMI index and increased CVD incidence in a population with CKM syndrome stage 0–3. This finding suggests that enhanced assessment of TyG-BMI index may provide a more convenient and effective tool for individuals at risk for CVD in CKM syndrome stage 0–3.

**Supplementary Information:**

The online version contains supplementary material available at 10.1186/s12933-024-02352-6.

## Introduction

In the Presidential Advisory issued by the American Heart Association (AHA) in October 2023, Cardiovascular-Kidney-Metabolic (CKM) syndrome was characterized as a systemic disorder manifesting through pathophysiological interactions among metabolic risk factors, chronic kidney disease (CKD), and the cardiovascular system. This interaction culminates in multiorgan dysfunction and a significantly elevated risk of adverse cardiovascular events [1]. An increasing body of research supports the concept of CKM syndrome, highlighting the intricate interactions among metabolic abnormalities, CKD, and cardiovascular diseases (CVD) [2–4]. Specifically, individuals with diabetes have a 2 to 4 times higher risk of heart failure compared to those without diabetes [5], and a nearly 40% prevalence of CKD has been documented [6]. Among the adult population in the United States, 5% concurrently suffer from cardiac, renal, and metabolic diseases, with this proportion on the rise [7]. It is notable that in the CKM staging framework, the AHA considers testing individuals in the preclinical stages to be of significant importance and emphasises that research on stages 0 to 3 CKM populations should focus on the prevention of CVD events [1]. Furthermore, substantial evidence indicates that the greatest clinical burden attributed to CKM syndrome is disproportionately related to CVD [8], underscoring the urgency of addressing metabolic, renal, and cardiovascular components as a unified system to prevent progression from stages 0–3 of CKM syndrome.

The triglyceride glucose-body mass index (TyG-BMI index), utilized as a measure for assessing insulin resistance (IR) [9, 10], has been correlated with CVD in numerous studies [11–14]. The TyG-BMI index, which is a composite index that combines triglyceride glucose index (TyG) and body mass index (BMI), has been demonstrated to markedly enhance the efficacy of IR assessment in comparison to the TyG index alone [15]. However, the association between the TyG-BMI index and CVD within a CKM syndrome population remains uncertain.

Therefore, given the importance of CKM syndrome in the development of cardiovascular disease, it is necessary to study the association between TyG-BMI and cardiovascular incidence in the population with CKM syndrome from stage 0 to stage 3 [16, 17], which will help the study of progressive cardiovascular disease and the implementation of early and comprehensive intervention, and provide a basis for reducing cardiovascular disease and the burden of disease in the population [1].

### Data source and study population

The data for this study were obtained from the China Health and Retirement Longitudinal Study (CHARLS). CHARLS is a national cohort study that targets Chinese adults aged 45 and above, conducting surveys periodically from 2011 to 2018. This study employed a multi-stage stratified probability-proportional-to-size sampling strategy, enrolling participants from both urban and rural areas across 28 provinces and 150 counties or districts in China. The design and cohort profile of the CHARLS study have been extensively documented in previous publications. The CHARLS study adhered to the principles of the Declaration of Helsinki and received approval from the Institutional Review Board at Peking University (IRB00001052-11015). All participants provided written informed consent prior to participating in the CHARLS study. In the CHARLS study, all fieldwork staff received systematic and professional training and conducted face-to-face interviews using standardized questionnaires [18]. In this study, participants interviewed from 2011 to 2012 were considered as the baseline, and followed up in the years 2013, 2015, and 2018.

**Fig. 1:**
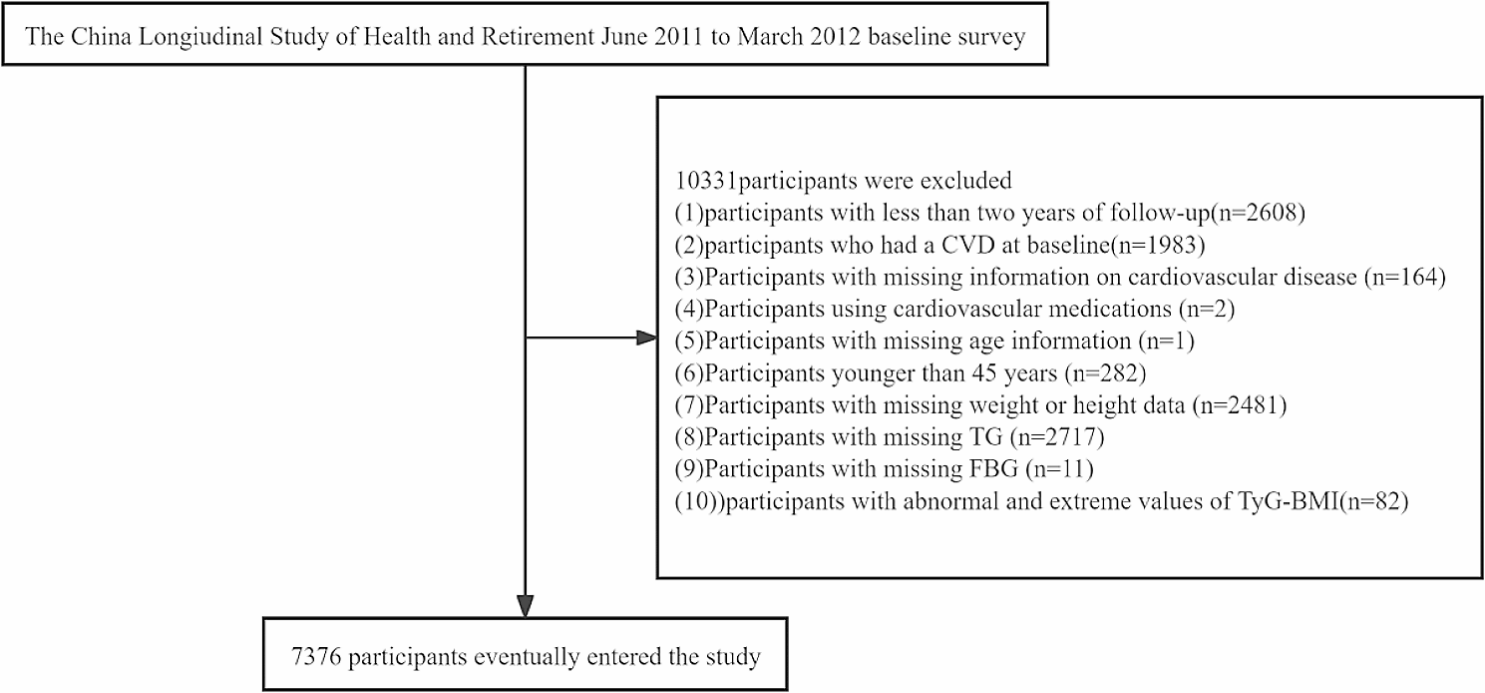
Flowchart of the study population

The flowchart (Fig. 1) delineates the inclusion and exclusion criteria of this study. Initially, we excluded 2608 participants who had follow-up periods of less than two years. Additionally, 1983 participants who had CVD at baseline, 164 participants missing information on CVD, and 2 participants using cardiovascular medications were also excluded. Furthermore, 1 participant missing age information and 282 participants younger than 45 years were excluded. Subsequently, we further removed 2481 participants lacking weight or height data, 2717 participants missing triglyceride (TG) data, and 11 participants without fasting plasma glucose (FBG) data. We also excluded 82 participants whose TyG-BMI values were beyond three standard deviations (SD) above the mean. Consequently, a total of 7376 participants were included in the analysis.

### Variables

#### Calculation of triglyceride glucose-body mass index

In this study, TyG-BMI was defined as follows: TyG-BMI = BMI × TyG index, where the TyG index = ln [FPG (mg/dL) × TG (mg/dL) / 2], and BMI is calculated as weight divided by the square of height (kg/m²) [19].

## CVD diagnosis

The primary endpoint of this study was the incidence of cardiovascular disease during the follow-up period (from Wave 2 to Wave 4). In alignment with previous related research, information regarding the historical diagnosis of CVD was collected using a standardized question: “Have you been diagnosed with [heart attack, coronary heart disease, angina, congestive heart failure, or other heart problems] by a doctor?“ [20]. The CHARLS study team implemented rigorous quality control measures for data recording and verification to ensure the reliability of the data [18].

## Definition of CKM syndrome stage 0 to 3

The stages of CKM Syndrome from 0 to 3 are categorized in accordance with the AHA Presidential Advisory Statement on CKM Syndrome [1]. The stages are delineated as follows: Stage 0 involves the absence of CKM syndrome risk factors; Stage 1 is characterized by excess or dysfunctional adiposity; Stage 2 includes metabolic risk factors or CKD; and Stage 3 encompasses subclinical cardiovascular disease. For the purpose of this classification, very high-risk CKD (stage G4 or G5 CKD) and a High predicted 10-year CVD risk by the Framingham risk score were employed as risk equivalents for subclinical CVD [21]. eGFR was calculated using the Chinese Modification of Diet in Renal Disease (C-MDRD) equation [22] and was classified into CKD stages according to Kidney Disease Improving Global Outcomes (KDIGO) [1].

### Data collection

The following data were collected for the purposes of this study:

(i) Demographic data: age, gender, education level, marital status.
(ii) Body measurements: systolic blood pressure (SBP), diastolic blood pressure (DBP), height, weight, and waist circumference.
(iii) Lifestyle data: smoking and drinking status, sleep problems.
(iv) Data on disease history and medication history: hypertension, hypertension medication, diabetes, diabetes medication, liver diseases, lung diseases, cancer.
(v) Laboratory test data: Glycated Hemoglobin A1c (HbA1C), FBG, TG, total cholesterol (TC), high-density lipoprotein cholesterol (HDL-c), low-density lipoprotein cholesterol (LDL-c), platelets (PLT), blood urea nitrogen (BUN), serum creatinine (Scr), C-reactive protein (CRP), uric acid (UA).

Those participants who reported a history of hypertension or were receiving specific treatment for hypertension, as well as those with an SBP of 130 mmHg or greater or a DBP of 80 mmHg or greater at baseline, were defined as hypertensive [23]. Individuals who reported a history of diabetes or were undergoing treatment for this condition, as well as those with an FBG of ≥ 7.0 mmol/L (126 mg/dL) or an HbA1c of ≥ 6.5% at baseline, were considered to have diabetes [24, 25]. Depression was defined using the 10-item short form of the Center for Epidemiologic Studies Depression Scale (CESD10) [26]. Participants with a total score ≥ 10 were identified as exhibiting depressive symptoms.Sleep quality was judged by the question “My sleep in the last week was restless.“, which had four options: little or no time (< 1 day), some of the time (1–2 days), occasional or moderate time (3–4 days), and most or all of the time (5–7 days). moderate time (3–4 days), and most or all of the time (5–7 days). Participants who answered > = 1 were identified as having sleep problem [27].Other medical statuses were determined by self-report.

### Handling of missing variables

Additional file: Table S1 illustrates the extent of data missingness in this study.Although the majority of variables exhibited only minor degrees of data incompleteness, multiple imputation was employed to preserve the largest possible sample size, thereby approximating more closely the true conditions [28].

### Statistical analysis

Participants in this study were divided into four groups (Q1-Q4) based on quartiles of TyG-BMI. For continuous variables that displayed a normal distribution, statistics were described using means ± SD, and differences between groups were inferred using analysis of variance (ANOVA). For continuous variables that did not follow a normal distribution, the median and interquartile ranges were utilized for statistical description, and group-wise differences were examined using the Kruskal-Wallis H test. Categorical variables were characterized by frequencies and percentages, with intergroup differences assessed using the χ² test. The relationship between TyG-BMI and the incidence of CVD was prospectively analyzed using univariate and multivariate Cox regression models. To explore the association between TyG-BMI and CVD incidence across different demographic characteristics, subgroup and interaction analyses were conducted among various age groups (< 60 vs. ≥ 60 years), genders, smoking statuses, drinking statuses, and CKM syndrome stages (Stage 0 to Stage 3). In the test for multicollinearity (Additional file: Table S2), the results showed that the variance inflation factor (VIF) for each covariate was less than 5, indicating that there was no evidence of significant multicollinearity between the covariates [29].To investigate the potential nonlinear relationship between TyG-BMI and CVD incidence, restricted cubic spline (RCS) regression of hazard ratio (HR) was employed in the total CKM syndrome stage 0 to 3 population, as well as in the CKM stage 2 and CKM stage 3 populations, respectively. In addition, sensitivity analyses were conducted on the data prior to multiple imputation to verify the robustness of the results (Additional file: Tables S3 and S4). All statistical analyses were conducted using R software (version 4.4.0), and two-sided P value < 0.05 was considered statistically significant.

## Results

### Baseline characteristics of participants

**Fig. 2:**
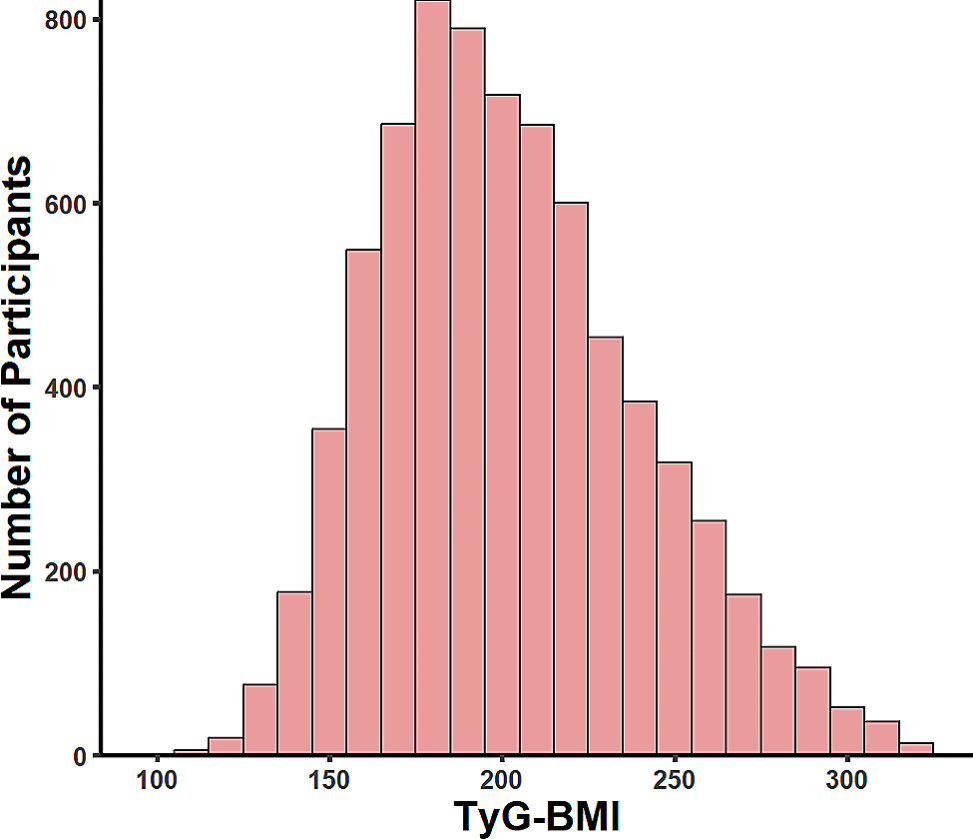
Distribution of TyG-BMI index

A total of 7376 participants were enrolled, of which 47.38% were females, with an average age of 59.17 ± 9.28 years. Baseline characteristics, as presented in Table 1 according to quartiles of TyG-BMI, reveal that the proportion of current smokers and drinkers was lower in the higher TyG-BMI groups. Additionally, these groups exhibited higher levels of PLT, TC, HbA1c, UA, all *P* < 0.05. Figure 2 illustrates the distribution of TyG-BMI, with a mean value of 202.30 ± 37.63 kg/m², indicating a normal distribution of TyG-BMI.

**Table 1:** Baseline characteristics of individuals classified by quartiles of the TyG-BMI index

Characteristic	Q1 (< 174.75)	Q2 (174.75-197.73)	Q3 (197.73-226.33)	Q4 (> 226.33)	*P*-value
Age, years	61.70 ± 9.86	59.32 ± 9.30	58.49 ± 8.94	57.16 ± 8.37	< 0.001
Female	1064(57.70)	926(50.22)	807(43.76)	698(37.85)	< 0.001
Married	1580(85.68)	1606(87.09)	1643(89.10)	1699(92.14)	< 0.001
Education level					< 0.001
No completion of primary school	986(53.47)	897(48.64)	856(46.42)	809(43.87)	
Sishu/home school/elementary school	431(23.37)	426(23.10)	414(22.45)	398(21.58)	
Middle school	294(15.94)	362(19.63)	366(19.85)	429(23.26)	
High school and above	133(7.21)	159(8.62)	208(11.28)	208(11.28)	
Waist circumference, cm	74.86 ± 8.60	80.59 ± 9.79	86 0.28 ± 9.75	93.81 ± 12.06	< 0.001
BMI, kg/m^2^	19.35 ± 1.62	22.00 ± 1.32	24.18 ± 1.54	27.55 ± 2.43	< 0.001
Systolic, mmHg	125.29 ± 21.17	128.05 ± 22.55	131.69 ± 24.61	136.08 ± 26.70	< 0.001
Diastolic, mmHg	71.89 ± 11.56	74.02 ± 11.67	76.41 ± 11.93	79.77 ± 12.01	< 0.001
Smoking statues					< 0.001
Never	908(49.24)	1,076(58.35)	1,189(64.48)	1,293(70.12)	
Former	141(7.65)	135(7.32)	161(8.73)	175(9.49)	
Current	795(43.11)	633(34.33)	494(26.79)	376(20.39)	
Drinking statues					< 0.001
Never	1139(61.77)	1174(63.67)	1215(65.89)	1314(71.26)	
Former	103(5.59)	90(4.88)	99(5.37)	108(5.86)	
Current	602(32.65)	580(31.45)	530(28.74)	422(22.89)	
Platelets, (×10^9/L)	208.82 ± 76.06	210.82 ± 71.71	210.02 ± 72.53	218.32 ± 83.93	< 0.001
BUN, mg/dl	16.32 ± 4.70	15.85 ± 4.71	15.46 ± 4.24	15.37 ± 4.35	< 0.001
FBG, mg/dl	99.62 ± 19.11	104.60 ± 27.11	109.88 ± 33.57	124.25 ± 48.91	< 0.001
Scr, mg/dL	0.78 ± 0.18	0.78 ± 0.32	0.78 ± 0.18	0.78 ± 0.20	0.800
TC, mg/dl	184.12 ± 35.39	190.82 ± 37.30	196.04 ± 37.58	203.05 ± 40.48	< 0.001
TG, mg/dl	79.97 ± 36.19	102.49 ± 47.50	132.77 ± 70.84	204.31 ± 144.83	< 0.001
HDL-c, mg/dl	60.06 ± 15.89	54.90 ± 14.43	49.07 ± 12.90	42.42 ± 11.75	< 0.001
LDL-c, mg/dl	109.86 ± 31.21	116.94 ± 32.89	120.78 ± 34.28	118.05 ± 40.31	< 0.001
CRP, mg/dl	2.82 ± 9.25	2.45 ± 6.78	2.42 ± 6.75	2.64 ± 4.55	< 0.001
HBA1C,%	5.10 ± 0.54	5.16 ± 0.68	5.23 ± 0.76	5.53 ± 1.07	< 0.001
UA, mg/dl	4.28 ± 1.19	4.32 ± 1.20	4.49 ± 1.26	4.68 ± 1.29	< 0.001
eGFR	123.98 ± 30.03	122.54 ± 28.90	120.65 ± 28.08	119.76 ± 32.37	< 0.001
TyG	8.19 ± 0.44	8.48 ± 0.45	8.76 ± 0.52	9.24 ± 0.66	< 0.001
TyG-BMI	158.23 ± 12.45	185.98 ± 6.63	211.13 ± 8.09	253.84 ± 21.36	< 0.001
Cancer	1822(98.81)	1835(99.51)	1833(99.40)	1824(98.92)	0.043
Lung diseases	1610(87.31)	1674(90.78)	1711(92.79)	1722(93.38)	< 0.001
Liver disease	1775(96.26)	1783(96.69)	1775(96.26)	1779(96.48)	0.900
Sleep problems	953(51.68)	982(53.25)	892(48.37)	877(47.56)	0.001
Depression	1617(87.69)	1628(88.29)	1596(86.55)	1617(87.69)	0.400
Hypertension	189(10.25)	273(14.80)	436(23.64)	667(36.17)	< 0.001
Diabetes	27(1.46)	54(2.93)	87(4.72)	180(9.76)	< 0.001
MetS	116(6.29)	358(19.41)	914(49.57)	1,523(82.59)	< 0.001
CVD	154(8.35)	191(10.36)	203(11.01)	285(15.46)	< 0.001
CKM stage					< 0.001
0	814(44.14)	308(16.70)	17(0.92)	0(0.00)	
1	393(21.31)	566(30.69)	425(23.05)	131(7.10)	
2	199(10.79)	379(20.55)	583(31.62)	678(36.77)	
3	438(23.75)	591(32.05)	819(44.41)	1035(56.13)	

Note: Data are presented as the mean (SD), median(quantile1,quantile3) or number (%), as appropriate

### The relationship between the TyG-BMI index and the incidence of CVD in a population with CKM syndrome stages 0–3

In this study, a total of 833 participants developed CVD, resulting in an incidence rate of 11.29%. To evaluate the association between TyG-BMI and CVD incidence among participants across CKM syndrome stages 0–3, five Cox proportional hazards models were developed (Table 2). Initially, Model I identified that for every 10-unit increase in TyG-BMI, there was a 6.7% increase in CVD risk (HR = 1.067, 95% CI: 1.049–1.086). In Model II, a 10-unit increase in TyG-BMI was associated with a 7.0% increase in risk (HR = 1.070, 95% CI: 1.051–1.089). Model III showed an 7.0% increase in risk for every 10-unit rise in TyG-BMI (HR = 1.070, 95% CI: 1.051–1.089). In Model IV, the association persisted with a 8.5% increase in risk per 10-unit increment in TyG-BMI (HR = 1.085, 95% CI: 1.061–1.109).Model V: For every 10-unit increase in TyG-BMI, there was a 6.5% increase in CVD risk (HR = 1.065, 95% CI: 1.041–1.090).

**Table 2:** Association between the TyG-BMI index and CVD incidence in a population with CKM syndrome stages 0–3

	Model I (HR., 95%CI)	*P*	Model II (HR., 95%CI)	*P*	Model III (HR., 95%CI)	*P*	Model IV (HR., 95%CI)	*P*	Model V (HR., 95%CI)	*P*
TyG-BMI (per10 units)	1.067(1.049 ~ 1.086)	< 0.001	1.069(1.051, 1.089)	< 0.001	1.070(1.051~1.089)	< 0.001	1.085(1.061~1.109)	< 0.001	1.065(1.041~1.090)	< 0.001
TyG-BMI quartile										
Q1	Ref		Ref		Ref		Ref		Ref	
Q2	1.256(1.016~1.553)	0.0358	1.282(1.036,1.587)	0.022	1.280(1.034~1.585)	0.023	1.300(1.047~1.615)	0.018	1.257(1.011~1.562)	0.040
Q3	1.333(1.081~1.644)	0.007	1.358(1.099,1.678)	0.005	1.352(1.093~1.672)	0.005	1.416(1.130~1.774)	0.002	1.294(1.031~1.625)	0.026
Q4	1.929(1.585~2.346)	< 0.001	1.977(1.617,2.417)	< 0.001	1.966(1.605~2.407)	< 0.001	2.129(1.682~2.694)	< 0.001	1.798(1.410~2.294)	> 0.001

MODEL 1: crude model

MODEL 2: adjusted for Age, Gender

MODEL 3: adjusted for Age, Gender, Smoking statues, Drinking statues, Sleep problems, Education level, Marital status

MODEL 4: adjusted for Age, Gender, Smoking statues, Drinking statues, Sleep problems, Education level, Marital status, BUN, Scr, TC, HDL-c, LDL-c, CRP, UA, PLT

MODEL5: adjusted for Age, Gender, Smoking statues, Drinking statues, Sleep problems, Education level, Marital status, BUN, Scr, TC, HDL-c, LDL-c, CRP, UA, PLT, Hypertension,Diabetes, Depression

Further clarifying the relationship between TyG-BMI and CVD incidence, TyG-BMI was categorized into quartiles. In the fully adjusted Model V, compared to the first quartile (Q1), the hazard ratio (HR) for Q2, Q3, and Q4 were 1.257 (95% CI: 1.011–1.562), 1.294 (95% CI: 1.031–1.625), and 1.798 (95% CI: 1.410–2.294), respectively. This indicates that participants in Q2, Q3, and Q4 experienced a 25.7%, 29.4%, and 79.8% higher risk of CVD compared to those in Q1 among the CKM syndrome stages 0–3 population.

To further explore the relationship between TyG-BMI and the incidence of CVD, subgroup and interaction analyses were conducted across different age groups, genders, smoking statuses, drinking statuses, and CKM syndrome stages (Stage 0 to Stage 3). The results (Table 3) showed interaction effects only among different age groups (P for interaction = 0.005), with no interactions observed in other subgroups (P for interaction > 0.05).

**Table 3:** Subgroup analyses of the association between the TyG-BMI index and CVD incidence in a population with CKM syndrome stages 0–3

Characteristics	Number of participants	HR (95%CI)	*P*	*P* for interaction
Age (years)				0.005
< 60	4102	1.080(1.045,1.116)	< 0.001	
≥ 60	3274	1.048(1.015,1.082)	0.004	
Gender				0.432
Female	3881	1.076(1.046,1.107)	< 0.001	
Male	3495	1.051(1.010,1.094)	0.014	
Smoking status				0.318
Never	4466	1.078(1.049,1.109)	< 0.001	
Ever	614	1.060(0.979,1.147)	0.152	
Current	2296	1.039(0.990,1.091)	0.119	
Drinking status				0.533
Never	4831	1.076(1.048,1.106)	< 0.001	
Ever	410	1.006(0.899,1.126)	0.916	
Current	2135	1.050(0.999,1.104)	0.053	
CKM stage				0.948
Stage 0	1140	1.043(0.912,1.193)	0.538	
Stage1	1515	1.062(0.989,1.141)	0.099	
Stage 2	1838	1.058(1.008,1.110)	0.023	
Stage 3	2883	1.058(1.008,1.110)	< 0.001	

The model was adjusted for Age, Gender, Smoking statues, Drinking statues, Sleep problems, Education level, Marital status, BUN, Scr, TC, HDL-c, LDL-c, CRP, UA, PLT, Hypertension, Diabetes, Depression; (excluding the variable for subgroup stratification)

**Fig. 3:**
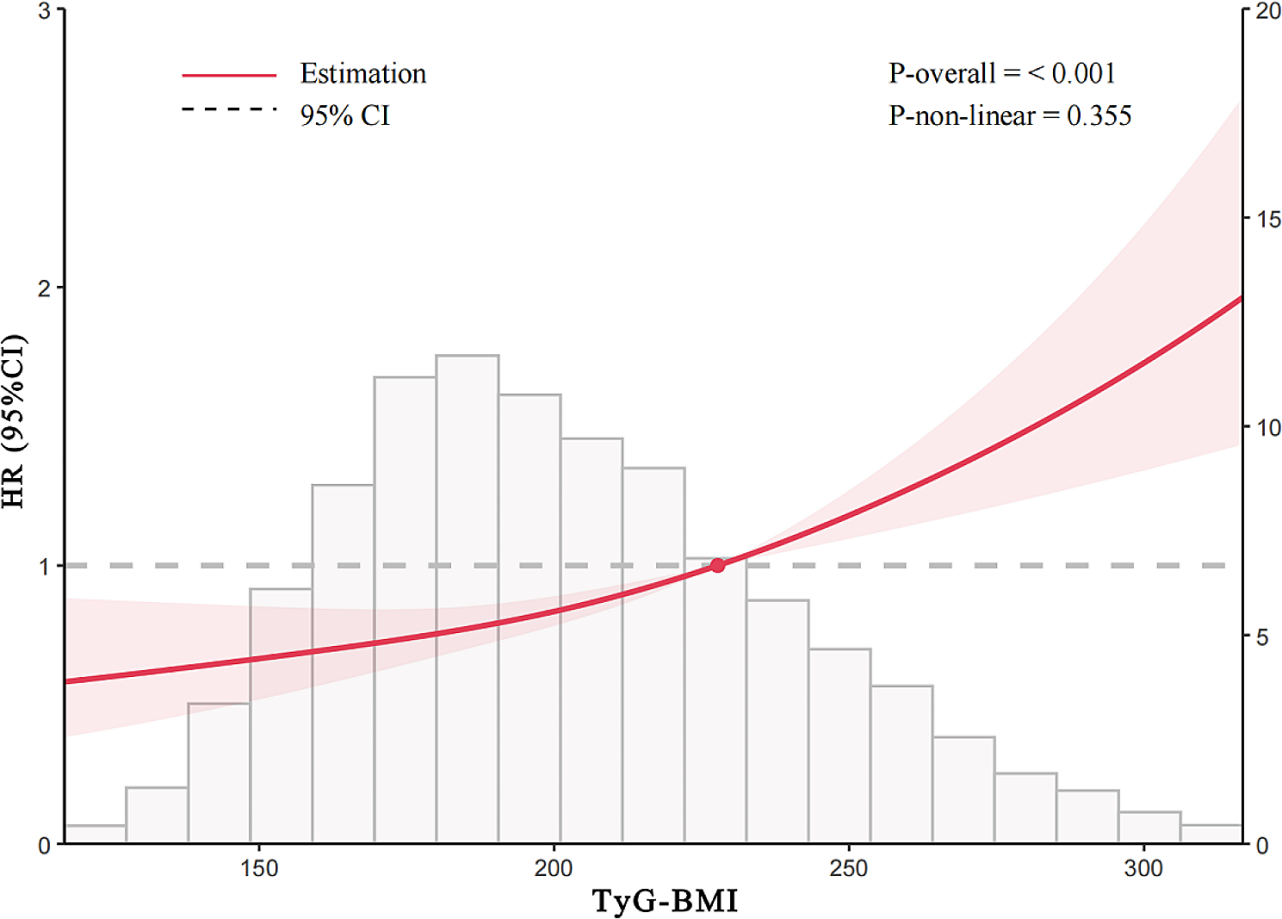
The RCS analysis between the TyG-BMI index and CVD incidence in a population with CKM syndrome stages 0–3. The model was adjusted for Age, Gender, Smoking statues, Drinking statues, Sleep problems, Education level, Marital status, BUN, Scr, TC, HDL-c, LDL-c, CRP, UA, PLT, Hypertension, Diabetes, Depression

**Fig. 4:**
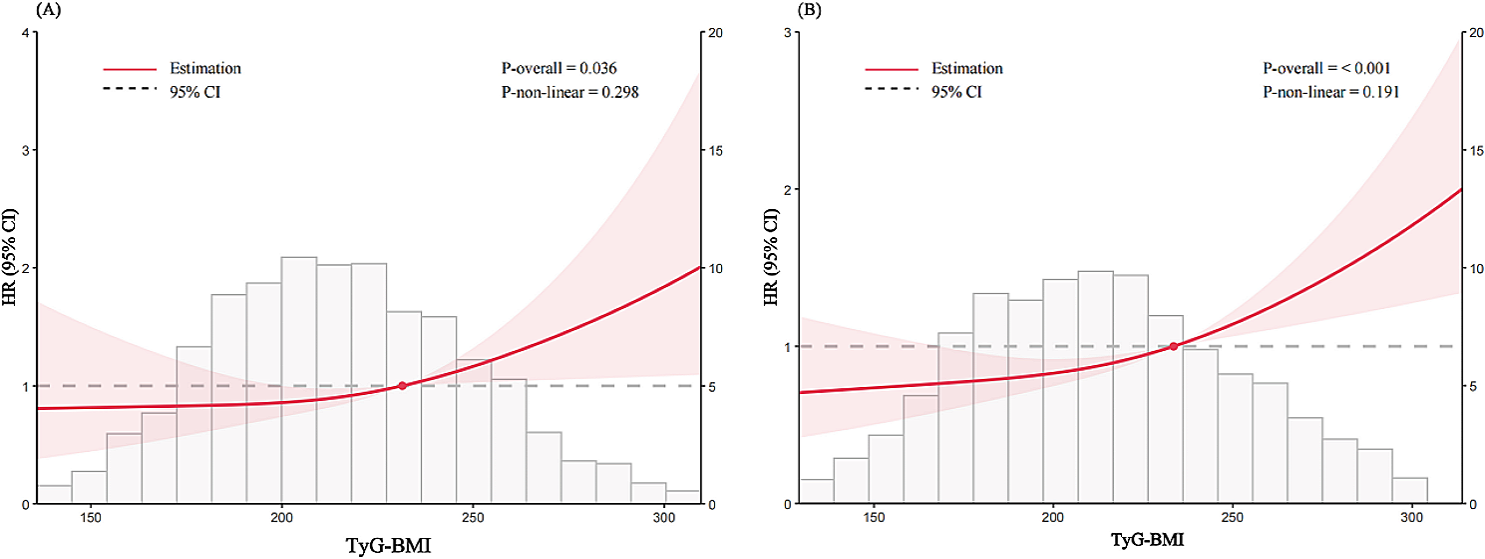
The RCS analysis between the TyG-BMI index and CVD incidence in a population with CKM syndrome stage 2 (**A**) or stage 3 (**B**). The model was adjusted for Age, Gender, Smoking statues, Drinking statues, Sleep problems, Education level, Marital status, BUN, Scr, TC, HDL-c, LDL-c, CRP, UA, PLT, Hypertension, Diabetes, Depression

Restricted cubic spline (RCS) Cox proportional hazard regression models were used to further examine the association between TyG-BMI and the risk of CVD among participants with CKM syndrome. The fully adjusted RCS regression model demonstrated (Fig. 3) a positive linear relationship between TyG-BMI and CVD risk among the overall participants in CKM syndrome stages 0–3 (P-overall < 0.001, P-non-linear = 0.355).Similarly, a positive linear relationship was observed between the TyG-BMI index and CVD risk in participants with CKM syndrome stage 2 (P-overall = 0.036, P-nonlinearity = 0.298) and stage 3 (P-overall < 0.001, P-nonlinearity = 0.191)(Fig. 4).

## Discussion

Based on the results of a literature search, this study is the first to explore the association between the TyG-BMI index and CVD in the context of CKM syndrome. Numerous studies have indicated a significant correlation between the TyG-BMI index and CVD in the general population [11, 30, 31]. Given the interactions between metabolic dysregulation, CKD, and CVD [16, 17], investigating the link between TyG-BMI and CVD in the backdrop of CKM syndrome is deemed essential.

IR refers to the decreased glucose uptake ability exhibited by insulin-sensitive tissues such as skeletal and cardiac muscles, adipose tissue, and the liver due to reduced biological effects of insulin [32]. Extensive research has identified IR as an independent risk factor for CVD [33–35]. The hyperinsulinemic-euglycemic clamp (HEC) is the gold standard for assessing IR, but its complex and costly technical requirements render it impractical in clinical settings [36]. Hence, identifying a reliable surrogate marker for broader IR assessment is vitally necessary.

The TyG-BMI index, recently developed as a measure of IR, has been widely used due to its high accuracy and ease of implementation [37, 38]. A study from CHARLS indicated that for each standard deviation increase in cumulative average TyG-BMI, the risk of CVD events increased by 16.8%, and this association was positively linear [11]. Another study from CHARLS suggested that every 10-unit increase in TyG-BMI augmented the risk of stroke by 4.9% [39]. A 10-year follow-up study comparing the ability of seven alternative IR indices to predict the risk of coronary heart disease found that the relative risk for the fifth quintile of new-onset coronary disease in relation to TyG-BMI was 3.169, higher than the other six indices [31].

Our findings reveal an association between the TyG-BMI index and CVD among participants in CKM syndrome stages 0–3. For every 10-unit increase in TyG-BMI, the risk of CVD incidence rose by 6.5%. Participants in the fourth TyG-BMI quartile of CKM syndrome had an approximately 1.80 times higher risk of CVD compared to those in the first quartile. More importantly, our study further corroborates that this association is linear. These results help elucidate the predictive value of the TyG-BMI index in the CKM syndrome population, enabling the more precise identification of high-risk individuals.

Our study also identified statistically significant interactions between different age groups, suggesting that managing the TyG-BMI in individuals aged 40 to 60 years could considerably lower the incidence of CVD compared to those older than 60 years.

The TyG-BMI index is a combination of TG, FBG and BMI levels to assess insulin resistance. Studies have shown that insulin resistance can lead to an imbalance in glucose metabolism resulting in hyperglycaemia, which in turn triggers inflammation and oxidative stress [40], which may contribute to the development of atherosclerosis [41]. Secondly, insulin resistance can impair endothelial function by inducing an increase in the production of glycosylation products and free radicals, leading to nitric oxide (NO) inactivation [42], and by inducing an overproduction of reactive oxidative stress (ROS) [43], which in turn may contribute to the development of cardiovascular disease. In addition, in the early stages of atherosclerosis, insulin resistance may also lead to an increase in atherosclerotic plaques through the downregulation of the insulin receptor-Akt1 signalling pathway, causing, among other things, a decrease in the activation of eNOS in arterial endothelial cells and an increase in the expression of VCAM-1 [44]. There is evidence that insulin resistance is also associated with the promotion of smooth muscle cell proliferation, which can lead to cardiac fibrosis and thus accelerate the onset of heart failure [45].The above studies show that, despite the complexity of the pathogenesis of cardiovascular disease, insulin resistance indices represented by TyG-BMI can explain some of it and provide ideas for the prevention and treatment of cardiovascular disease.

Our results showed no significant statistical correlation between the TyG-BMI index and the incidence of CVD in CKM syndrome stages 0 and 1, possibly due to these individuals not having significant metabolic or cardiovascular risk factors or not yet reaching a substantial threshold. In contrast, in individuals with higher metabolic risk factors at CKM syndrome stages 2 and 3, the association between the TyG-BMI index and CVD incidence was magnified, consistent with previous findings [46]. Notably, our results indicated that the interaction among different CKM syndrome stage 0–3 groups was not statistically significant, underscoring the need for more extensive, prospective cohort studies to clarify whether the association between TyG-BMI and CVD incidence is consistent across all stages of CKM syndrome. Apart from different age groups, no significant variations were observed in the relationship between TyG-BMI and the incidence of cardiovascular diseases within other subgroups (gender, smoking status, drinking status and CKM stage). This suggests the generalizability of our study findings to a broad population.

The advantages of this study are clear: firstly, it is a prospective, large-sample cohort study that evaluated the association between TyG-BMI and CVD within the CKM syndrome population for the first time and explored the form of this association. Secondly, we employed multiple imputation to address missing covariate data, enhancing the statistical power and reliability of our analyses. Thirdly, we analyzed the TyG-BMI index as both categorical and continuous (per 10 units) variables to assess its associations with CVD risk, enabling identification of risk differences across various TyG-BMI levels and aligning with clinical realities.Fourthly, we also explored the form of the association between the TyG-BMI index and CVD risk in the CKM syndrome population, identifying a positive linear relationship, and found that this relationship still exists in CKM stages 2 and 3. Fifthly, subgroup analysis revealed specific subgroups within the CKM syndrome population where managing the TyG-BMI index could significantly reduce CVD risk, and sensitivity analyses were conducted to evaluate the robustness of our findings.

However, the limitations of our study should not be overlooked. Firstly, the person-time calculation used an approximation method, introducing potential errors, though similar approaches have been fitted with COX regression [20]. Secondly, in defining subclinical CVD, we did not use the latest PREVENT equations but applied the Framingham 10-year cardiovascular risk score, which has been validated and widely used in Asian populations [47]. Thirdly, the diagnosis of CVD was self-reported by participants in CHARLS, which may slightly deviate from the actual incidence rates. Fourthly, our study only included middle-aged and elderly Chinese individuals; thus, generalizing the findings may be limited. Finally, as a single-center study, despite using multivariate adjustments and subgroup analyses, potential confounding factors may still be missed.

## Conclusion

This cohort study demonstrated an association between the TyG-BMI index and an increased incidence of CVD within the population at CKM syndrome stages 0–3. Notably, this association presented as a linear relationship, which indicates that the enhanced evaluation of the TyG-BMI index could provide a more convenient and effective means of screening for individuals at high risk of CVD among those with CKM syndrome stages 0–3.

### Electronic supplementary material

Below is the link to the electronic supplementary material.

Supplementary Material 1

## Data Availability

The datasets generated and/or analysed during the current study are available in the China Health and Retirement Longitudinal Study repository [http://charls.pku.edu.cn].

## Abbreviations

AHA: American Heart Association
CKM: Cardiovascular-Kidney-Metabolic
CKD: Chronic kidney disease
CVD: Cardiovascular diseases
TyG-BMI: Triglyceride glucose-body mass index
IR: Insulin resistance
TyG: Triglyceride glucose index
BMI: Body mass index
TG: Triglyceride
FBG: Fasting plasma glucose
BMI: Body mass index
SD: Standard deviations
C-MDRD: Chinese Modification of Diet in Renal Disease
KDIGO: Kidney Disease Improving Global Outcomes
SBP: Systolic blood pressure
DBP: Diastolic blood pressure
HbA1c: Glycated Hemoglobin A1c
TC: Total cholesterol
HDL-c: High-density lipoprotein cholesterol
LDL-c: Low-density lipoprotein cholesterol
PLT: Platelets
BUN: Blood urea nitrogen
Scr: Serum creatinine
CRP: C-reactive protein
UA: Uric acid
CESD10: 10-item short form of the Center for Epidemiologic Studies Depression Scale
ANOVA: Analysis of variance
VIF: Variance inflation factor
RCS: Restricted cubic spline
HR: Hazard ratio
CI: Confidence interval
HEC: Hyperinsulinemic-euglycemic clamp
ROS: Reactive oxygen species
