# Supplementary material for: Association between the triglyceride glucose-body mass index and future cardiovascular disease risk in a population with Cardiovascular-Kidney-Metabolic syndrome stage 0–3: a nationwide prospective cohort study
Source: Cardiovasc Diabetol. 2024 Aug 7;23:292. doi: 10.1186/s12933-024-02352-6 (PMC11308445; doi:10.1186/s12933-024-02352-6)
Supplement: Supplementary file 1 — Supplementary Material 1 [file 12933_2024_2352_MOESM1_ESM.pdf]

Table S1. Distribution of variables with missing data

variables	Number of Missing	Missing proportion
Drinking statues	590	8.00%
Platelets	132	1.79%
Diastolic	80	1.08%
Systolic	68	0.92%
HBA1C	57	0.77%
Diabetes	50	0.68%
Liver Disease	28	0.38%
Cancer	23	0.31%
Smoking statues	23	0.31%
Waist circumference	16	0.22%
Hypertension	15	0.20%
Lung Diseases	11	0.15%
LDL-c	8	0.11%
Gender	5	0.07%
BUN	1	0.01%
Scr	1	0.01%

Table S2. Collinearity Statistics.

Variables	GVIF	Df	$GVIF^{1/2Df}$
TyG-BMI	1.740006	1	1.319093
Age	1.372595	1	1.171578
Gender	2.708285	1	1.645687
Smoking status	2.093511	2	1.202870
Drink status	1.557207	2	1.117086
Sleep problem	1.053759	1	1.026528
Education level	1.286569	3	1.042891
Marital status	1.121119	1	1.058829
BUN	1.157016	1	1.075647
Scr	1.492893	1	1.221840
TC	3.846017	1	1.961126
HDL-c	1.525082	1	1.234942
LDL-c	3.362668	1	1.833758
CRP	1.017593	1	1.008758
UA	1.475594	1	1.214740
PLT	1.040723	1	1.020158
Hypertension	1.134848	1	1.065292
Diabetes	1.045534	1	1.022514
Depression	1.033249	1	1.016489

Table S3. Baseline characteristics after excluding individuals with any missing values.

Characteristic	Q1 (<174.71)	Q2 (174.71-197.71)	Q3 (197.71-226.10)	Q4 (>226.10)	*P*-value
Age,years	61.84±9.9	59.41±9.27	58.43±8.9	57.19±8.24	<0.001
Female	900(56.71%)	773(48.71)	685(43.16)	572(36.04)	<0.001
Married	1,360(85.70)	1,390(87.59)	1,426(89.86)	1,469(92.56)	<0.001
Education level					<0.001
No completion of primary school	866(54.57)	785(49.46)	735(46.31)	698(43.98)	
Sishu/home school/elementary school	370(23.31)	378(23.82)	367(23.13)	344(21.68)	
Middle school	253(15.94)	293(18.46)	323(20.35)	366(23.06)	
High school and above	98(6.18)	131(8.25)	162(10.21)	179(11.28)	
Waist circumference,cm	75.01±8.29	80.79±9.22	86.47±8.88	94.1±11.24	<0.001
BMI,kg/m^2	19.37±1.56	21.96±1.33	24.15±1.52	27.56±2.38	<0.001
Systolic,mmHg	125.51±21.22	127.93±20.3	131.45±22.42	135.65±21.07	<0.001
Diastolic,mmHg	71.91±11.56	74.04±11.66	76.45±12.04	79.78±11.92	<0.001

**Characteristic**	**Q1 (<174.71)**	**Q2 (174.71-197.71)**	**Q3 (197.71-226.10)**	**Q4 (>226.10)**	***P*-value**
Smoking statues					<0.001
Never	788(49.65)	935(58.92)	1,038(65.41)	1,137(71.64)	
Former	120(7.56)	117(7.37)	135(8.51)	146(9.20)	
Current	679(42.79)	535(33.71)	414(26.09)	304(19.16)	
Drinking statues					<0.001
Never	977(61.56)	1,013(63.83)	1,045(65.85)	1,142(71.96)	
Former	88(5.55)	84(5.29)	84(5.29)	91(5.73)	
Current	522(32.89)	490(30.88)	458(28.86)	354(22.31)	
Platelets,(×10^9/L)	210.19±76.93	210.65±71.62	209.53±72.78	218.25±85.69	0.003
BUN,mg/dl	16.35±4.73	15.88±4.79	15.48±4.21	15.32±4.36	<0.001
FBG,mg/dl	99.91±19.15	104.94±27.92	109.71±31.84	123.72±46.07	<0.001
Scr,mg/dL	0.78±0.18	0.78±0.34	0.77±0.18	0.77±0.2	0.6
TC,mg/dl	184.79±35.44	191.53±37.54	196.35±38.1	202.78±39.44	<0.001
TG,mg/dl	79.51±35.43	102.75±47.17	133.07±71.31	198.36±124.5	<0.001

**Characteristic**	**Q1 (<174.71)**	**Q2 (174.71-197.71)**	**Q3 (197.71-226.10)**	**Q4 (>226.10)**	***P*-value**
HDL-c,mg/dl	60.55±15.98	55.13±14.39	49.55±13.1	42.7±11.38	<0.001
LDL-c,mg/dl	110.04±31.49	117.33±33.31	120.55±34.63	118.66±39.15	<0.001
CRP,mg/dl	2.83±9.41	2.55±7.18	2.49±7.13	2.68±4.58	<0.001
HBA1C,	5.12±0.56	5.17±0.7	5.23±0.74	5.52±1.02	<0.001
UA,mg/dl	4.26±1.19	4.31±1.2	4.49±1.28	4.62±1.28	<0.001
eGFR	123.97±30.1	122.52±29.39	120.96±28.32	119.91±31	<0.001
TyG	8.19±0.42	8.48±0.45	8.76±0.52	9.22±0.64	<0.001
TyG-BMI	158.43±12.02	185.82±6.62	210.91±8.04	253.59±21.39	<0.001
Cancer	1,575(99.24)	1,580(99.56)	1,580(99.56)	1,569(98.87)	0.057
Lung Diseases	1,386(87.33)	1,432(90.23)	1,475(92.94)	1,486(93.64)	<0.001
Liver Disease	1,527(96.22)	1,529(96.35)	1,524(96.03)	1,532(96.53)	0.9
Sleep problems	812(51.17)	850(53.56)	759(47.83)	756(47.64)	0.001
Depression	1,397(88.03)	1,402(88.34)	1,378(86.83)	1,391(87.65)	0.6
Hypertension	170(10.71)	231(14.56)	358(22.56)	575(36.23)	<0.001

Characteristic	Q1 (<174.71)	Q2 (174.71-197.71)	Q3 (197.71-226.10)	Q4 (>226.10)	*P*-value
Diabetes	26(1.64)	49(3.09)	69(4.35)	157(9.89)	<0.001
MetS	99(6.24)	313(19.72)	787(49.59)	1,323(83.36)	<0.001
CVD	134(8.44)	174(10.96)	176(11.09)	254(16.01)	<0.001
CKM stage					<0.001
0	688(43.35)	257(16.19)	9(0.57)	0(0.00)	
1	342(21.55)	476(29.99)	375(23.63)	111(6.99)	
2	166(10.46)	316(19.91)	500(31.51)	561(35.35)	
3	391(24.64)	538(33.90)	703(44.30)	915(57.66)	

Note:Data are presented as the mean (SD) ,median(quantile1,quantile3) or number (%), as appropriate.

Table S4. Association between the TyG-BMI index and CVD incidence in a population with CKM syndrome stages 0-3 after excluding individuals with any missing value.

	Model I (HR., 95%CI)	*P*	Model II (HR., 95%CI)	*P*	Model III (HR., 95%CI)	*P*	Model IV (HR., 95%CI)	*P*	Model V (HR., 95%CI)	*P*
TyG-BMI (per10 units)	1.069(1.050~1.089)	<0.001	1.070(1.051~1.091)	<0.001	1.069(1.049~1.090)	<0.001	1.088(1.063~1.114)	<0.001	1.070(1.044,1.097)	<0.001
TyG-BMI quartile										
Q1	Ref		Ref		Ref		Ref		Ref	
Q2	1.317(1.051~1.650)	0.017	1.335(1.064~1.674)	0.012	1.325(1.056~1.662)	0.015	1.352(1.073~1.703)	0.010	1.318(1.046,1.661)	0.019
Q3	1.325(1.058~1.659)	0.014	1.343(1.070~1.685)	0.011	1.339(1.065~1.682)	0.012	1.418(1.113~1.805)	0.005	1.323(1.037,1.687)	0.024
Q4	1.972(1.599~2.431)	<0.001	1.994(1.609~2.470)	<0.001	1.963(1.580~2.437)	<0.001	2.164(1.680~2.786)	<0.001	1.863(1.436,2.418)	<0.001

MODEL1:Crude model

MODEL2:Adjusted for Age, Gender;

MODEL3:Adjusted for Age, Gender, Smoking statues, Drinking statues, Sleep problems, Education level, Marital status;

MODEL4:Adjusted for Age, Gender, Smoking statues, Drinking statues, Sleep problems, Education level, Marital status, BUN, Scr, TC, HDL-c, LDL-c, CRP, UA, PLT;

MODEL5:Adjusted for Age, Gender, Smoking statues, Drinking statues, Sleep problems, Education level, Marital status, BUN, Scr, TC, HDL-c, LDL-c, CRP, UA, PLT, Hypertension, Diabetes, Depression;
